# Diet-related urine collections: assistance in categorization of hyperoxaluria

**DOI:** 10.1007/s00240-021-01290-2

**Published:** 2021-11-25

**Authors:** Hannah Dill, Cristina Martin-Higueras, Bernd Hoppe

**Affiliations:** 1 Department of Pediatrics, Division of Pediatric Nephrology, University Hospital Bonn, Bonn, Germany; 2 Department of Ciencias Médicas Básicas, Faculty of Medicina, Universidad de La Laguna, San Cristóbal de La Laguna, Tenerife Spain; 3 German Hyperoxaluria Center, Im Mühlenbach 2b, 53127 Bonn, Germany

**Keywords:** Secondary hyperoxaluria, Primary hyperoxaluria, Oxalate, Diet, Urine analysis

## Abstract

Hyperoxaluria, one of the major risk factors for calcium oxalate urolithiasis and nephrocalcinosis, causes significant morbidity and mortality and should therefore be detected and treated as soon as possible. An early, consequent and adequate evaluation, but also a distinction between primary (PH) and secondary hyperoxaluria (SH) is therefore essential. We evaluated the usefulness of three consecutive 24-h urine collections under different diets [usual diet, (A), low oxalate diet, (B), high oxalate diet, (C)] to prove SH, or to find evidence of PH by changes in urinary oxalate excretion (Uox). We retrospectively analyzed results from 96 pediatric patients (47 females and 49 males, age 3–18 years) who presented with a history of nephrolithiasis, nephrocalcinosis and/or persistent hematuria in whom hyperoxaluria was found in an initial urine sample. The typical pattern of SH was found in 34 patients (mean Uox (A) 0.85 ± 0.29, (B) 0.54 ± 0.15 and (C) 0.95 ± 0.28 mmol/1.73m^2^/d). PH was suspected in 13 patients [(A) 1.21 ± 0.75; (B) 1.47 ± 0.51 and (C) 1.60 ± 0.82 mmol/1.73m^2^/d], but genetically proven only in 1/5 patients examined. No hyperoxaluria was found in 16 patients. Data were inconclusive in 33 patients. Urine collection under different diets is helpful to diagnose secondary hyperoxaluria and may provide evidence, that urinary oxalate excretion is normal. We have now established this procedure as our first diagnostic step before further, more extensive and more expensive evaluations are performed.

**Supplementary Information:**

The online version contains supplementary material available at 10.1007/s00240-021-01290-2.

## Introduction

Hyperoxaluria, defined as urinary oxalate excretion > 0.5 mmol/1.73m^2^/d, is one of the major risk factors for stone formation in both children and adults*.* Hyperoxaluria is divided into primary and secondary forms. Three types of primary hyperoxaluria (PH) are currently known, all based on enzymatic defects of glyoxylate metabolism in the liver (Alanine:glyoxylate aminotransferase, AGT, in PH I, glycolate reductase:hydroxypyruvate reductase, GRHPR, in PH II and Hydroxy-oxo-glutarate aldolase type 1, HOGA-1, in PH III) [1–3]. All of them can lead to endogenous oxalate overproduction [4–6]. As oxalate is an end product of human metabolism, it is primarily eliminated via the kidneys, where it forms stones or induces the development of nephrocalcinosis.

Secondary, dietary or enteric, hyperoxaluria (SH) has different causes. Dietary SH is usually caused by increased intake of oxalate rich foods or oxalate precursors (e.g., ascorbic acid). *Oxalobacter formigenes*, an anaerobic bacterium normally colonizing the intestinal tract and utilizing oxalate as its sole source of energy, degrades dietary oxalate intestinally. A lack of Oxalobacter leads to an increased intestinal oxalate availability and consecutively increased absorption [4, 7–10]. An increased intestinal absorption is also found in patients with malabsorption syndromes, chronic inflammatory bowel diseases (e.g., Crohn´s disease or cystic fibrosis), short bowel syndromes and in patients after gastric bypass surgeries [11–15]. Moreover, defects of the intestinal oxalate transporters (e.g., SLC26A6) may induce hyperoxaluria [16].

Untreated PH as well as SH can lead to recurrent nephrolithiasis and progressive nephrocalcinosis. In severe cases, end-stage renal disease (ESRD) occurs early and leads to systemic oxalate deposition especially in PH type I, but also very dramatically in patients with Crohn’s disease especially after ileocecal valve resection [4, 11]. It is therefore of utmost interest to detect hyperoxaluria, but also to differentiate its reasons as early as possible to initiate proper treatment and to prevent irrevocable long-term consequences [6, 14]. In the contrary, diagnosis is often delayed. For example, more than 30% of patients with PH I already suffer from ESRD or had a failed isolated kidney transplantation when finally diagnosed [17].

Gold standard to detect hyperoxaluria, as well as any other lithogenic risk factor, is the repeated examination of 24-h urine collections [18]. Nevertheless, the differentiation between PH and SH can still be problematic. Here, the determination of further urinary metabolites found in PH (glycolate in PH I, glyceric-acid in PH II and hydroxy-oxo-glutarate or dihydroxy-glutarate in PH III), analysis of plasma oxalate, the [^13^C_2_] oxalate absorption test, stool tests for *Oxalobacter formigenes* or even microbiota testing and finally genetic workup to detect variants in the different PH genes (AGXT-/GHPR-/HOGA 1) are possible diagnostic procedures [3, 4, 7, 19–22]. This may turn out to be a long-lasting and expensive process, which in total is not routinely applicable, especially in children (e.g., an inpatient [^13^C_2_] oxalate absorption testing).

Studies have shown that diet has a reasonable influence on intestinal oxalate absorption and hence on urinary oxalate excretion [9, 23, 24]. Holmes et al. showed a significant contribution of dietary oxalate to urinary oxalate excretion (Uox) even in healthy individuals [9]. Uox peaks 2 to 4 hours after the oxalate ingestion and after 24-h dietary oxalate is mostly excreted [25, 26].

We aimed to improve the diagnostic procedure for hyperoxaluria by evaluating three 24-h urine collections under different dietary regimen in an outpatient setting. This might then be a helpful tool to (1) identify secondary hyperoxaluria, (2) find evidence of primary hyperoxaluria or (3) exclude hyperoxaluria.

## Subjects and methods

The ambulatory 3 × 24-h urine collection under different dietary regimen was introduced into our routine diagnostic program in 2007 after a study with an inpatient [^13^C_2_] oxalate absorption test was found unfeasible in a pediatric setting [22]. We now retrospectively analyzed pediatric patient data documented from 07/2007 to 02/2020. Patients were either routinely examined at our center, or their urine samples (collected according to our protocol) were sent to our laboratory from other clinics. The urine testings were done because of a history of nephrocalcinosis and/or (calcium-oxalate) nephrolithiasis and/or hematuria as well as hyperoxaluria in a single 24-h urine collection beforehand. Patients with already proven primary hyperoxaluria (no further testing necessary), or patients with malabsorption or chronic inflammatory bowel diseases, or post intestinal resections were not considered eligible. It is well known, that they either only have a minor (PH) or a hugely increased intestinal oxalate absorption compared to healthy subjects (SH) [11, 12, 22].

Three 24-h urine collections were performed on consecutive days under a normal/usual diet without specific dietary regulation (the patients were asked to maintain their usual dietary habits, day 1), a low oxalate diet (day 2) and a high oxalate diet (day 3). A high oxalate load in the diet (roughly 600 mg oxalate) was ensured by consumption of oxalate-rich foods such as spinach (e.g., 150 g for lunch), rhubarb (e.g., 500 ml rhubarb juice), sweet potatoes, nuts, chocolate, beetroot and parsley. Supplemental Tables 1 and 2 provide an overview of the recommended diets and an exemplary nutrition protocol for a 12-year-old girl, respectively. To avoid carryover of diet high in oxalate from day 1 to day 2 with the low oxalate diet, patients had to avoid oxalate-rich foods post lunch. In addition, it was assured that calcium intake was according to the German daily recommended allowances (depending on age: 600–1200 mg/day) and hence not influencing the oxalate absorption profoundly. The patients were encouraged to avoid these oxalate-rich foods during urine collection under the low oxalate diet, resulting in a reduced oxalate intake of about one-tenth (63 mg) of the oxalate content of the high oxalate diet. Furthermore, fluid intake had to remain constant and any supplement, e.g., ascorbic acid intake as an oxalate precursor, was forbidden. Patients had to summarize dietary and fluid intakes on a spreadsheet provided (Supplemental Table 3). Precise instructions and the importance of adhering to the dietary requirements were summarized by a specialized dietician and provided to the patients together with the urine collection protocol (Supplemental Tables 1 and 2). Adapted to the outpatient setting, we have kept the dietary advices as easy as possible to ensure the patients compliance.

For preservation of the urine samples, 10 ml of thymol 5% in isopropanol was added per liter expected urine volume before collections. Two urine aliquots (out of the total urine collected) of 10 ml each were sent to our laboratory, and one of the samples was then directly acidified to a pH of < 3 (but not < 1.5) for the following oxalate analysis.

Besides oxalate, glycolate (PH I), calcium and citrate were determined under all dietary conditions (Table 1). Urinary creatinine was analyzed in each urine to determine the adequacy of the urine collection. Calcium and creatinine were measured photometrically (by VIS-photometry)*.* For measurement of oxalate, citrate, glycolate and glyceric acid we used an ion chromatography/mass spectrometry (IC/MS) system [28]. Urinary hydroxy-oxo-glutarate analysis was performed retrospectively in all stored urines (before 2014) and later as routine procedure with an adapted IS/MS method [29].

**Table 1:** Mean oxalate, citrate, calcium and creatinine values ± SD of the different groups (1–4) under usual/normal diet, low oxalate diet and high oxalate diet

Oxalate diet	Usual (mean ± SD)	Low (mean ± SD)	High (mean ± SD)
All patients (*n* = 96; male = 49, mean age = 8.8 ± 4.3 years)			
Oxalate [mmol/1.73m^2^/d]	0.75 ± 0.45	0.65 ± 0.42	0.79 ± 0.53
Citrate [mmol/1.73m^2^/d]	2.73 ± 1.51	2.71 ± 1.54	2.90 ± 1.51
Calcium [mg/kg/d]	2.73 ± 2.08	2.79 ± 2.08	2.71 ± 2.08
Creatinine adjusted for body weight [mg/kg/d]	20.08 ± 5.33	21.10 ± 8.07	19.28 ± 5.93
Group 1—SH (*n* = 34; male = 17, mean age = 7.9 ± 3.7 years)			
Oxalate [mmol/1.73m^2^/d]	0.85 ± 0.29	0.54 ± 0.15	0.95 ± 0.28
Citrate [mmol/1.73m^2^/d]	3.05 ± 1.78	2.85 ± 1.48	3.42 ± 1.42
Calcium [mg/kg/d]	2.96 ± 2.08	2.45 ± 1.97	2.81 ± 2.14
Creatinine adjusted for body weight and urine volume [mg/kg/d]	20.56 ± 4.26	20.23 ± 4.45	21.05 ± 5,80
Group 2—suspected PH (*n* = 13; male = 6, mean age = 7.4 ± 2.4 years)			
Oxalate [mmol/1.73m^2^/d]	1.21 ± 0.75	1.47 ± 0.51	1.60 ± 0.82
Glycolate [mmol/1.73m^2^/d]	1.42 ± 2.34	1.34 ± 1.43	1.13 ± 1.27
Citrate [mmol/1.73m^2^/d]	3.07 ± 1.33	2.75 ± 1.51	2.98 ± 1.80
Calcium [mg/kg/d]	2.54 ± 1.94	2.71 ± 1.92	2.41 ± 1.94
Creatinine adjusted for body weight and urine volume [mg/kg/d]	19.43 ± 4.30	19.61 ± 3.49	18.96 ± 4.54
Group 3—inconclusive (*n* = 33, male = 20, mean age = 9.7 ± 4.7 years)			
Oxalate [mmol/1.73m^2^/d]	0.67 ± 0.34	0.58 ± 0.25	0.52 ± 0.13
Citrate [mmol/1.73m^2^/d]	2.48 ± 1.24	2.75 ± 1.64	2.61 ± 1.29
Calcium [mg/kg/d]	2.69 ± 2.42	3.31 ± 2.45	2.77 ± 2.20
Creatinine adjusted for body weight and urine volume [mg/kg/d]	20.65 ± 6.53	23.34 ± 12.50	18.51 ± 6.13
Group 4—NH (*n* = 16, male = 6, mean age = 9.8 ± 5.0 years)			
Oxalate [mmol/1.73m^2^/d]	0.34 ± 0.12	0.36 ± 0.08	0.33 ± 0.12
Citrate [mmol/1.73m^2^/d]	2.30 ± 1.45	2.31 ± 1.51	2.42 ± 1.65
Calcium [mg/kg/d]	2.61 ± 1.61	2.49 ± 1.40	2.47 ± 2.08/ 2.63 ± 1.99
Creatinine adjusted for body weight and urine volume [mg/kg/d]	18.36 ± 5.79	19.64 ± 4.21	17.05 ± 6.42

Glycolate is additionally listed in cases of suspected PH (group 2)

Shortly, samples were diluted 100 × with 0.20 M boric acid solution. The IC/MS system (ICS 3100 und MSQ + , ThermoFisher, USA) was equipped with an AS11 high efficiency column as analytical phase and an AG11 guard column as stationary phase (both ThermoFisher, USA). For calibration five standards of increasing concentrations were used. The MS was calibrated to a span of 0.30 *m*/*z* at 161 *m*/*z*, negative polarity, dwell time of 0.5 s, cone voltage of 25 V, and a probe temperature of 450ºC for optimal detection. A potassium hydroxide (KOH) gradient (ThermoFisher, 3 KOH eluent cartridge) of 5 mM gradually ramping up to 100 mM over 38 min was used in the IC.

Based on the courses of the three oxalate values, we grouped the patients as follows:

Group 1 (SH), patients with elevated oxalate levels of > 0.5 mmol/1.73m^2^/d under high oxalate diet and an increase of > 20% from the low oxalate diet (day 2) to the normal/usual oxalate diet (day 3). This threshold was (intentionally) set above the 10–15% range of variation in urinary oxalate excretion described in repeated routine urine analyses. [30, 31] Adequate urine collection was assumed if the creatinine value adjusted to body weight and urine volume was between 11–26 mg/kg/day and was constant over the 3 days of testing (Table 1) [30, 32].

Follow-up data were available in *n* = 20 of the SH patients. Group 2 (suspected PH), patients with persistent hyperoxaluria of > 0.7 mmol/1.73m^2^/d on all days without showing any dietary influence [27, 33]. If available, data of genetic testing were also evaluated in this group (*n* = 5). Follow-up data were available in *n* = 7 patients. Group 3 (inconclusive), patients in whom no definitive interpretation of Uox was possible. Group 4 (no hyperoxaluria—NH), patients with normal oxalate excretion in all urines.

### Statistical analysis

For statistical analysis and data handling, we used JASP (version 0.14), R (version 3.5.0, R Foundation for Statistical Computing) and Microsoft Excel (version 16.29.1). Continuous variables are presented as means ± standard deviation. Categorial data are given as frequencies. For comparing the different oxalate diets in a single group, the repeated measures ANOVA Test was used. For comparing the individual groups with each other on all days (between-groups analysis), the ANOVA Test was used. The Bonferroni correction was used for post hoc testing. Sphericity was tested with Mauchly’s test and (if violated) corrected with the Greenhouse–Geisser method. A *p* value of < 0.05 was considered as statistically significant.

## Results

Adequacy of all urine collections was proven by urinary creatinine excretion (Table 1). Analyzing the oxalate values of the whole cohort (*n* = 96, 47 females and 49 males, age 3–18 years), mean Uox was above the hyperoxaluric threshold of 0.5 mmol/1.73m^2^/d under all diets (Table 1, Supplemental Fig. 1). The highest mean value (0.79 ± 0.53 mmol/1.73m^2^/d) was found at high oxalate diet and the lowest (0.65 ± 0.42 mmol/1.73m^2^/d, Table 1) at low oxalate diet. The mean Uox value under the usual diet was 0.75 ± 0.45 mmol/1.73m^2^/d. Mean oxalate excretion was significantly higher under the normal (*p* = 0.03) and high oxalate diets as compared to low oxalate diet (*p* = 0.002, Supplemental Fig. 1). There was no significant difference in Uox between the usual and the high oxalate diets (in all patients).

**Fig. 1:**
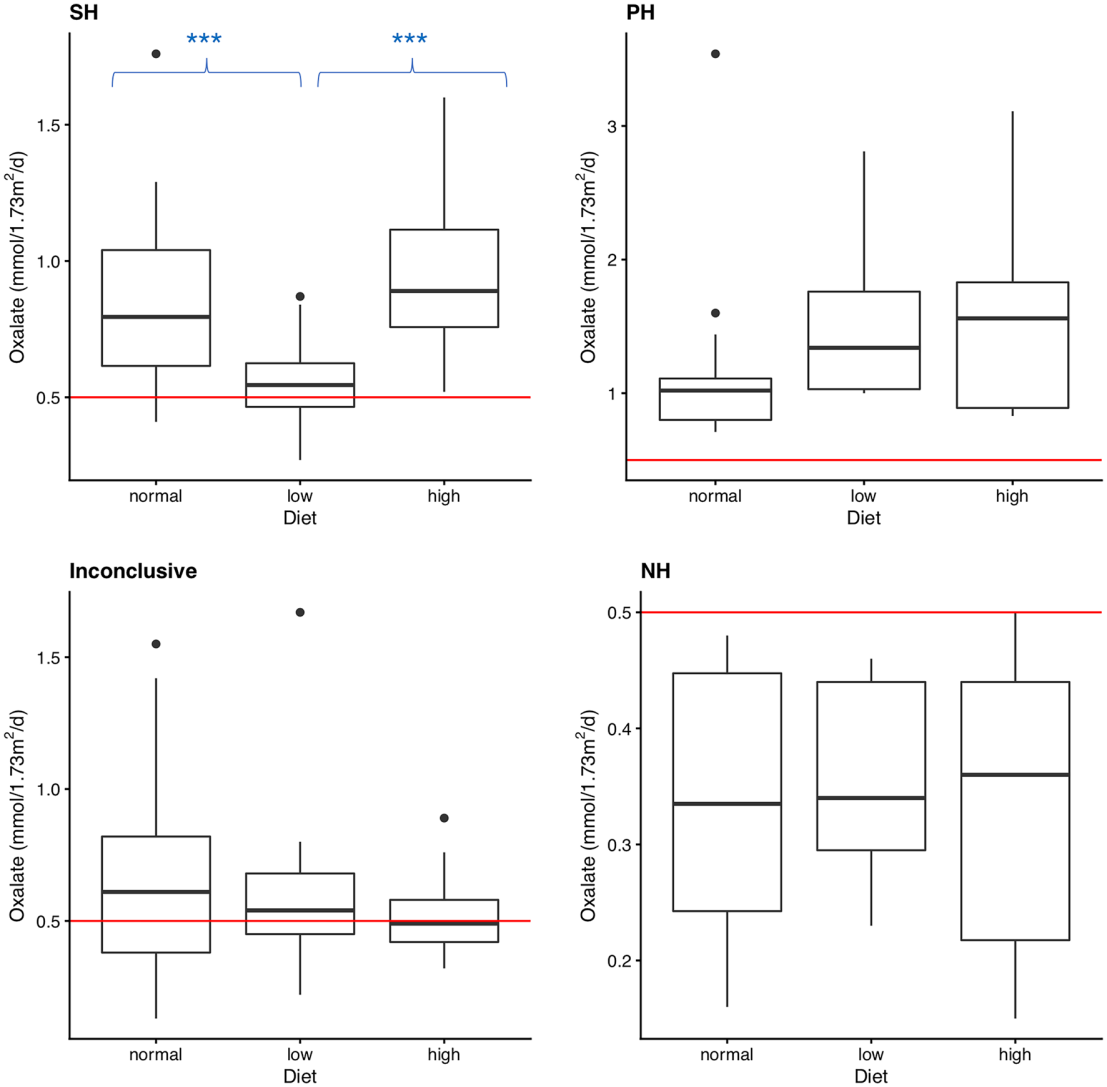
Boxplots of the oxalate values of the individual groups under usual/normal diet (day 1), low oxalate diet (day 2) and high oxalate diet (day 3). The horizontal line marks the hyperoxaluria threshold of 0.5 mmol/1.73m^2^/d. (Group 1: secondary hyperoxaluria (SH), group 2: suspected primary hyperoxaluria (PH), group 3: inconclusive, group 4: no hyperoxaluria (NH); *p ≤ 0.05; **p ≤ 0.01; ***p ≤ 0.001)

Urinary oxalate excretion showed the typical pattern of secondary hyperoxaluria in 34 patients (group 1, SH). Mean oxalate was 0.85 ± 0.29 mmol/1.73m^2^/d under usual diet, 0.54 ± 0.15 mmol/1.73m^2^/d under low and 0.95 ± 0.28 mmol/1.73m^2^/d under high oxalate diet (Table 1). It differed significantly for usual versus low oxalate diet (*p* < 0.001) and low versus high oxalate diet (*p* < 0.001), but not between normal/usual and high oxalate diet (*p* = 0.115, Fig. 1). Normal urinary oxalate values (< 0.5 mmol/1.73m^2^/d) were found in 13 of the 34 patients (38%) under low-oxalate diet (day 2). In 20/34 patients we were able to collect follow-up data under a low oxalate diet. Urinary oxalate values normalized and remained normal in 13/20 patients under the prescribed diet. In 3/20 patients urinary oxalate decreased to values > 0.5 ≤ 0.6 mmol/1.73m^2^/d. In 4/20 patients, follow data showed still elevated urinary oxalate > 0.6 mmol/1.73m^2^/d.

In 13 patients (group 2, PH), urinary oxalate excretion was constantly elevated on a high level (usual diet: 1.21 ± 0.75 mmol/1.73m^2^/d, low oxalate diet: 1.47 ± 0.51 mmol/1.73m^2^/d, high oxalate diet: 1.60 ± 0.82 mmol/1.73m^2^/d, Table 1) and did not differ significantly between collections (*p* = 0.061, Fig. 1). In addition to elevated oxalate levels, nine patients (69%) also had elevated glycolate levels. Hence, primary hyperoxaluria type 1 was suspected and genetic testing was recommended, but was only done in five patients. In one patient, PH 1 was diagnosed (*AGXT* variants). Further testing for mutations in the *GRHPR* (PH II) and the *HOGA1* gene (PH III) was performed in the other four patients, although neither urinary glyceric acid, nor hydroxy-oxo-glutarate was elevated in any of the patients.

Urinary oxalate excretion was not conclusive in 33 patients (group 3, inconclusive). Mean oxalate excretion was highest under the usual diet (0.67 ± 0.34 mmol/1.73m^2^/d), but only little changes were seen under the low (0.58 ± 0.25 mmol/1.73m^2^/d) as compared to the high oxalate diet (0.52 ± 0.13 mmol/1.73m^2^/d, Table 1). Accordingly, the diet showed no significant influence on the oxalate levels in group 3 (*p* = 0.071, Fig. 1).

**Fig. 2:**
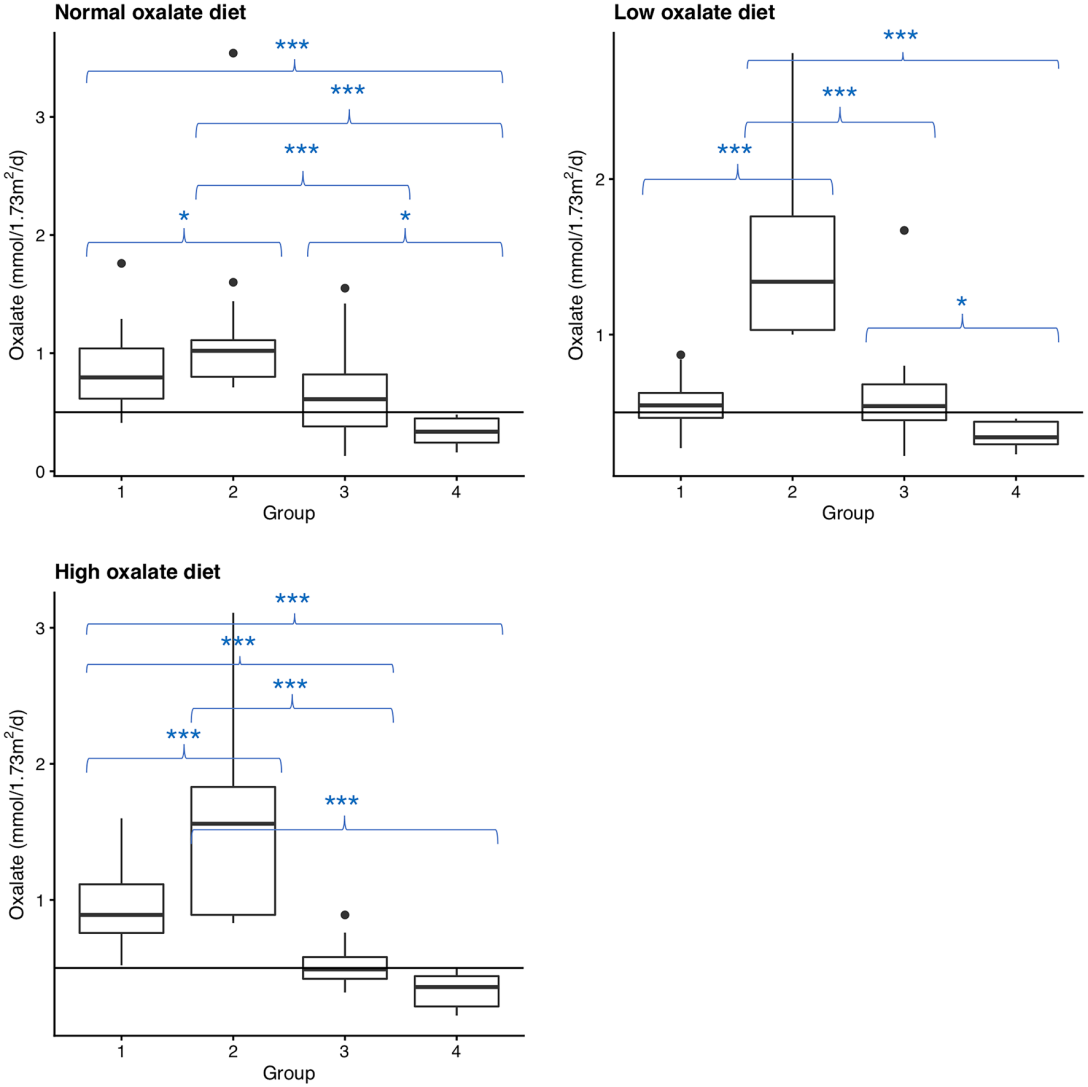
Boxplots of the oxalate excretion of the different groups under the different dietary conditions depicting significant differences between groups according to diet. The horizontal line marks the hyperoxaluria threshold of 0.5 mmol/1.73m^2^/d (Group 1: secondary hyperoxaluria (SH), group 2: suspected primary hyperoxaluria (PH), group 3: inconclusive, group 4: no hyperoxaluria (NH); *p ≤ 0.05; **p ≤ 0.01; ***p ≤ 0.001)

Urinary oxalate was not elevated and not significantly different (*p* = 0.698) between diets in 16 patients (group 4 (NH), usual diet: 0.34 ± 0.12 mmol/1.73m^2^/d, low oxalate diet: 0.36 ± 0.08 mmol/1.73m^2^/d, high oxalate diet: 0.33 ± 0.12 mmol/1.73m^2^/d), Table 1; Fig. 1). Hence, hyperoxaluria was excluded as the lithogenic risk factor (Fig. 2).

## Discussion

The three-time 24-h urine collection under different dietary regimen allows the classification of patients with suspected hyperoxaluria into different oxalate profiles, which facilitates diagnostic evaluation. Based on this, further diagnostic steps (if necessary at all) are possible in a more targeted manner. The procedure is particularly useful to identify the following two entities: first, the patients without hyperoxaluria can clearly be filtered out and secondly, those with the typical pattern of secondary hyperoxaluria can be identified. Hence, in 17% (16/96, group 4) we did not find hyperoxaluria with this testing, and we identified secondary hyperoxaluria in 35% (34/96, group 1) of the patients examined.

In SH patients, urinary oxalate levels were significantly influenced by the diet (Fig. 1). A usual diet depends on dietary habits and accordingly can vary in its oxalate content. The SH patients showed the second highest oxalate excretion under their usual diet (compared to the other days), indicating a particularly high oxalate content in their daily diet, which has to be considered for treatment advice. This is further underlined by significantly lower levels of urinary oxalate or even normal Uox (in 13/34 patients) achieved under the low oxalate diet (day 2). Therefore, next to hyperhydration, a low oxalate diet is the treatment of choice. Doing so, 16/20 patients, in whom long term follow up data were available, showed significantly lower or even normal oxalate levels.

No change in Uox based on dietary intervention was observed in group 2 (suspected PH). Constantly and significantly elevated urinary oxalate excretion, also accompanied by elevated glycolate levels in 69% (9/13) of the patients, let us suspect primary hyperoxaluria [29]. As determination of urinary glyceric acid (PH II) and hydroxy-oxo-glutarate (PH III), were only later started on a routine basis (2012/2016, respectively), we did not report these values in detail. However, we did not find elevated levels in any of the urines analyzed.

Of course, it would have been interesting to gain more information on long term follow up, especially in patients with secondary hyperoxaluria under a low oxalate diet. The test, however, was developed for outpatient use. So, we have received more than half of the urines from outside our clinic. Here, we were able to get clinical data as reason for the testing and in some patients we also received follow-up data. But we were only able to provide recommendations for further diagnostic evaluation. At time of analysis, we had to recognize that these recommendations were not taken in all cases. This, of course, has limited our data interpretation, especially on long-term outcome.

Since patients with PH can show a disastrous course of the disease, early diagnosis is mandatory and hence, genetic testing should be performed [6, 34, 35]. Data on genetic examination in all three PH genes, however, was only available in the 5 (out of 13) patients who were followed in our outpatient clinic. Primary hyperoxaluria type 1 was detected by AGXT variants in one patient. Nevertheless, further diagnostic evaluation in the other patients is needed and was now again recommended. The four patients without a mutation in a PH gene, but still significantly hyperoxaluric and clinically symptomatic, will now be included into an exome analysis project (in order) to detect rare variants with strong effects, thus potentially identifying novel forms of monogenic PH.

It was recently described (contrary to current opinion) that patients with PH may benefit from a restriction of food with extremely high oxalate content [36]. In our analysis, we only observed a slight increase in oxalate levels (of ≤ 15%) under the oxalate-rich diet, which is, however, in the range of the normal variability of oxalate levels in urine [30, 31, 37]. This does not answer the question of the necessity of an oxalate reduced diet in PH patients, but may indicate, that other treatment options (hyperhydration, B6 and citrate medication) are more beneficial. To improve compliance, we would not recommend a strict diet low in oxalate in patients with confirmed PH.

With only one patient diagnosed with PH, we also need to consider that patients did not adhere to the dietary protocol and still ate an extremely oxalate-rich diet during the collection days. This is a frequent experience and even if patients or parents were adequately instructed, surprising information on daily diets are often only committed after frequent questioning, or after a genetic testing result was negative. Such mal-compliance to test procedures may also be the reason why results were not concisely interpretable in about one third of all patients (33/96–group 3).

Nevertheless, in patients with inconclusive data or still unclear etiology (group 3 and 4) further diagnostic evaluation of other lithogenic risk factors are necessary. Hypocitraturia and/or hypercalciuria are the two main other risk factors of calcium-oxalate stones. A constantly low fluid intake should also be regarded as a major risk factor of stone disease (especially in young girls) causing supersaturation of urine and thus increasing the risk of nephrolithiasis, nephrocalcinosis and/or hematuria [38].

Often, however, long-term follow-up shows an amelioration of urinary compositions. Simply increasing fluid intake and explaining a low oxalate diet often results in complete normalization of urinary parameters and no further stone event in long-term follow-up observations.

## Conclusion

The ambulatory 3 × 24-h urine testing under different dietary regimen is a helpful tool to exclude hyperoxaluria and to diagnose secondary hyperoxaluria. Despite some limitations, it can serve as a clinical decision aid for evaluating patients with hyperoxaluria, before expensive and/or invasive diagnostics are performed.

Further research is needed to determine whether the procedure presented, may prove to be a valid test method in the future.

## Supplementary Information

Below is the link to the electronic supplementary material.

Supplementary S-Table 1: overview of the recommended diets for the three specific testing days (usual/normal, low and high oxalate diet). (DOCX 18 KB)

Supplementary S-Table 2: Exemplary nutrition protocol for girls aged 12 years and 9 months with different levels of oxalate intake (DOCX 16 KB)

Supplementary S-Table 3: Patients had to summarize dietary and fluid intakes on a spreadsheet provided (DOCX 69 KB)

Supplementary S-Fig. 1 Mean oxalate values of all patients (n = 96) under usual/normal diet (day 1), low oxalate diet (day 2) and high oxalate diet (day 3). The horizontal line marks the hyperoxaluria threshold of 0.5 mmol/1.73m2/d; * p ≤ 0.05; ** p ≤ 0.01; *** p ≤ 0.001 (PNG 48 kb)

Supplementary S-Fig. 1 Descriptive Plot showing the mean oxalate values of all groups under usual/normal, low and high oxalate diet. The horizontal line marks the hyperoxaluria threshold of 0.5 mmol/1.73m2/d (group 1: secondary hyperoxaluria (SH), group 2: suspected primary hyperoxaluria (PH), group 3: inconclusive, group 4: no hyperoxaluria (NH)) (PNG 80 kb)

## Data Availability

Not applicable.
