# Supplementary material for: Diet-related urine collections: assistance in categorization of hyperoxaluria
Source: Urolithiasis. 2021 Nov 25;50(2):141–8. doi: 10.1007/s00240-021-01290-2 (PMC8956551; doi:10.1007/s00240-021-01290-2)
Supplement: Supplementary file 1 — Supplementary S-Table 1: overview of the recommended diets for the three specific testing days (usual/normal, low and high oxalate diet). (DOCX 18 KB) [file 240_2021_1290_MOESM1_ESM.docx]

**Day 1 (24 h): patients usual diet (example)**

| Breakfast: | mixed bread with butter and jam/honey and some soft fruit with natural yogurt. |
| --- | --- |
| Lunch: | spaghetti with Bolognese sauce and carrot-pea vegetables |
| Afternoon: | fruit salad or butter cookies/lemon cake/sand cake/cheesecake |
| Dinner: | Mixed bread with butter and sausage/cheese and some raw vegetables (cucumbers/tomatoes/peppers) |
| Drinks: | Drinking water |
|  | |
| **Day 2 (24 h): low oxalate diet** | |
| Breakfast: | natural yogurt with 1 piece of fruit (apple/banana/watermelon/pineapple) and honey. |
| Lunch: | Rice (polished) with meat/fish and some cooked cauliflower or kohlrabi with cream sauce or cucumber salad or iceberg lettuce (please do not use fresh or dried herbs, such as parsley or dandelion). |
| Snack: | Fruit yogurt or 1-2 (depending on age) scoops of milk ice cream (without chocolate/without nuts) or 1 piece of fruit (watermelon, apple, pineapple) |
| Dinner: | 1 small slice of rye bread with butter and sausage/cheese and a small salad (of iceberg lettuce, cucumbers, tomatoes or peppers) and 1 glass of cow's milk. |
| Drinks: | Drinking water |
|  | |
| **Day 3 (24h): high oxalate diet** | |
| Breakfast: | Wholemeal bread/mixed bread with chocolate cream/nut cream and cocoa and fruit of choice Or chocolate muesli with milk (plus wheat bran or amaranth) and fruit of choice |
| Lunch: | Spinach or chard or beet vegetables with sweet potatoes and egg |
| Snacks: | Chocolate cake or nut cake or dark chocolate or rhubarb cake |
| Dinner: | Wholemeal bread/mixed bread with chocolate cream/nut cream/sesame paste and 1 glass of juice and 1 handful of nuts (e.g. almonds, hazelnut, cashew) Or chocolate muesli with milk (plus wheat bran or amaranth) and fruit of choice |
| Drinks: Drinking water Additional drinks possible according to age and preference: Black tea, concentrated peppermint tea | |
|  | |
