# Supplementary material for: Diet-related urine collections: assistance in categorization of hyperoxaluria
Source: Urolithiasis. 2021 Nov 25;50(2):141–8. doi: 10.1007/s00240-021-01290-2 (PMC8956551; doi:10.1007/s00240-021-01290-2)
Supplement: Supplementary file 2 — Supplementary S-Table 2: Exemplary nutrition protocol for girls aged 12 years and 9 months with different levels of oxalate intake (DOCX 16 KB) [file 240_2021_1290_MOESM2_ESM.docx]

|  | **Breakfast** | **Lunch** | **Afternoon Snack** | **Dinner** |
| --- | --- | --- | --- | --- |
| **„Usual“ oxalate intake** | 60 g of mixed bread with butter,  25 g jam,  200 g yogurt with 3.5% fat,  100 g strawberries | 220 g cooked spaghetti,  60 g bolognese sauce and one teaspoon parmesan cheese,  200 g carrots-peas-vegetables | 330 g fruit salad of apple, banana, strawberries, watermelon | 120 g mixed bread with butter,  40 g gouda cheese,  20 g poultry sausage,  150 g raw vegetable salad with yogurt dressing |
| **Nutrient intake: 2022 kcal, 134 mg oxalate, 1134 mg calcium** | | | | |
|  | **Breakfast** | **Lunch** | **Afternoon Snack** | **Dinner** |
| **Low oxalate intake** | 220 g yogurt 3.5 % fat,  90 g apple,  70 g banana,  25 g honey | 230 g cooked rice,  50 ml béchamel sauce,  120 g roasted poultry meat, 200 g iceberg lettuce with shredded carrots and vinegar-oil dressing | 200 g fruit yogurt,  30 g wafer cookie | 100 g rye bread with butter,  40 g gouda cheese,  20 g poultry sausage,  150 g raw vegetable salad with yogurt dressing |
| **Nutrient intake: 1988 kcal, 56 mg oxalate, 1164 mg calcium** | | | | |
|  | **Breakfast** | **Lunch** | **Afternoon Snack** | **Dinner** |
| **High oxalate intake** | 35 g chocolate oatmeal honey muesli puffed with amaranth, 90 g apple, 250 ml cow's milk 3.5% fat | 150 g spinach,  a dash of cream,  200 g potatoes,  fried egg | 150 g cheese-nut cake,  a tablespoon of whipped cream | 50 g wholemeal bread,  25 g chocolate cream,  60 g rye bread, 25 g cream cheese  150 g raw vegetables, a little cottage cheese dip, 10 g nuts, 100 ml juice |
| **Nutrient intake: 2068 kcal, 982 mg oxalate, 953 mg calcium** | | | | |
