# Supplementary material for: Diet-related urine collections: assistance in categorization of hyperoxaluria
Source: Urolithiasis. 2021 Nov 25;50(2):141–8. doi: 10.1007/s00240-021-01290-2 (PMC8956551; doi:10.1007/s00240-021-01290-2)
Supplement: Supplementary file 3 — Supplementary S-Table 3: Patients had to summarize dietary and fluid intakes on a spreadsheet provided (DOCX 69 KB) [file 240_2021_1290_MOESM3_ESM.docx]

**Dietary protocol for collecting urine for lithogenic substances -low oxalate, high oxalate-diet.**

| **Name:** | **Height: cm** |
| --- | --- |
| **First name:** | **Weight: Kg** |
| **Date of birth:** | **Gender: m f** |
| **Total urine volume: ml** |  |

**Please record everything you/your child eats and drinks during the collection period.**

**Date Day 1: ____________________ low oxalate diet**

| **Time** | **Diet** | **Fluid intake (ml)** |
| --- | --- | --- |
|  |  |  |
|  |  |  |
|  |  |  |
|  |  |  |
|  |  |  |
|  |  |  |
|  |  |  |
|  |  |  |
|  |  |  |
|  |  |  |
|  |  |  |
|  |  |  |

**Date Day 2: ____________________ high oxalate diet**

| **Time** | **Diet** | **Fluid intake (ml)** |
| --- | --- | --- |
|  |  |  |
|  |  |  |
|  |  |  |
|  |  |  |
|  |  |  |
|  |  |  |
|  |  |  |
|  |  |  |
|  |  |  |
|  |  |  |
|  |  |  |
|  |  |  |
