# Supplementary figures and images for: Diet-related urine collections: assistance in categorization of hyperoxaluria
Source: Urolithiasis. 2021 Nov 25;50(2):141–8. doi: 10.1007/s00240-021-01290-2 (PMC8956551; doi:10.1007/s00240-021-01290-2)

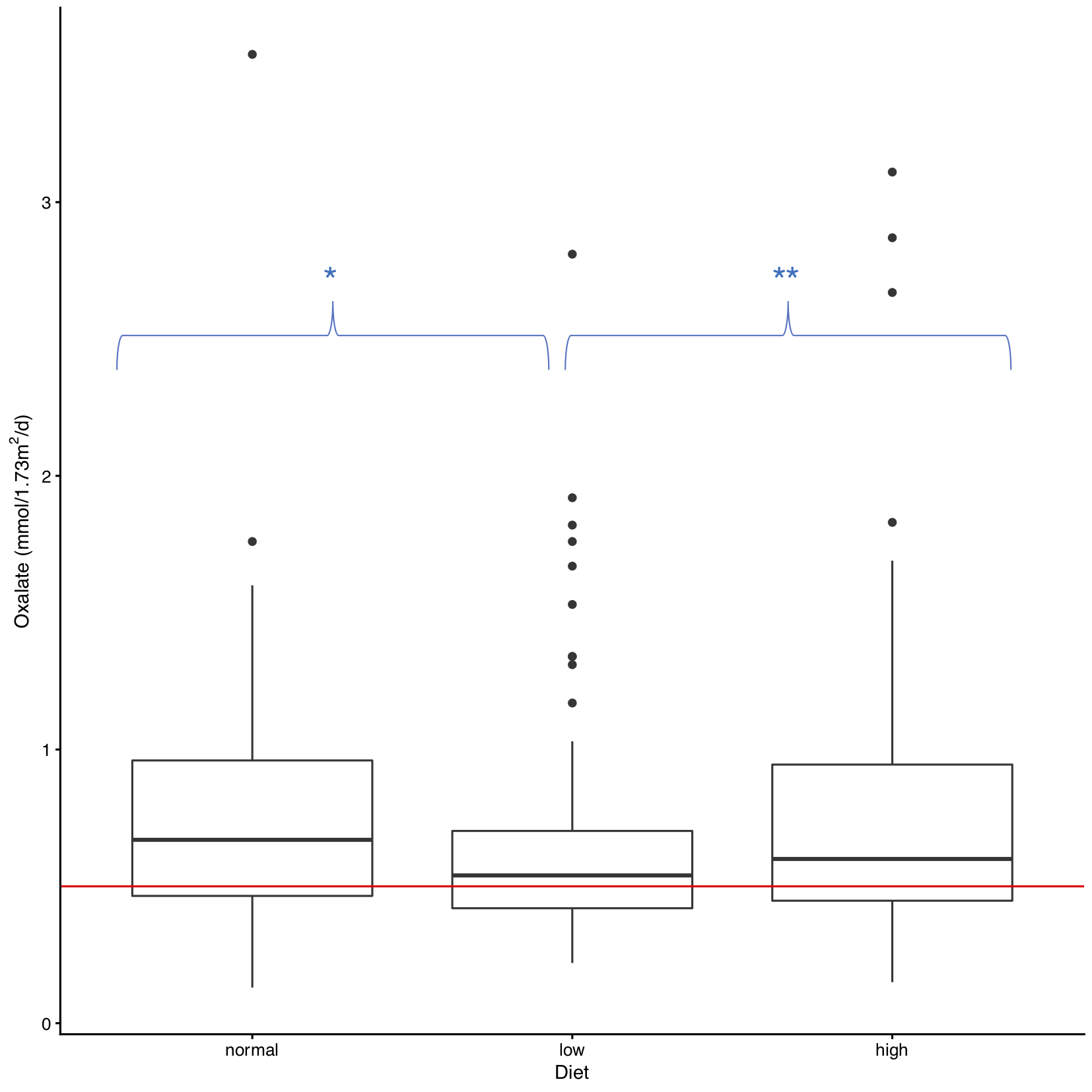

Supplement: Supplementary file 4 — Supplementary S-Fig. 1 Mean oxalate values of all patients (n = 96) under usual/normal diet (day 1), low oxalate diet (day 2) and high oxalate diet (day 3). The horizontal line marks the hyperoxaluria threshold of 0.5 mmol/1.73m2/d; * p ≤ 0.05; ** p ≤ 0.01; *** p ≤ 0.001 (PNG 48 kb) [file 240_2021_1290_MOESM4_ESM.png]

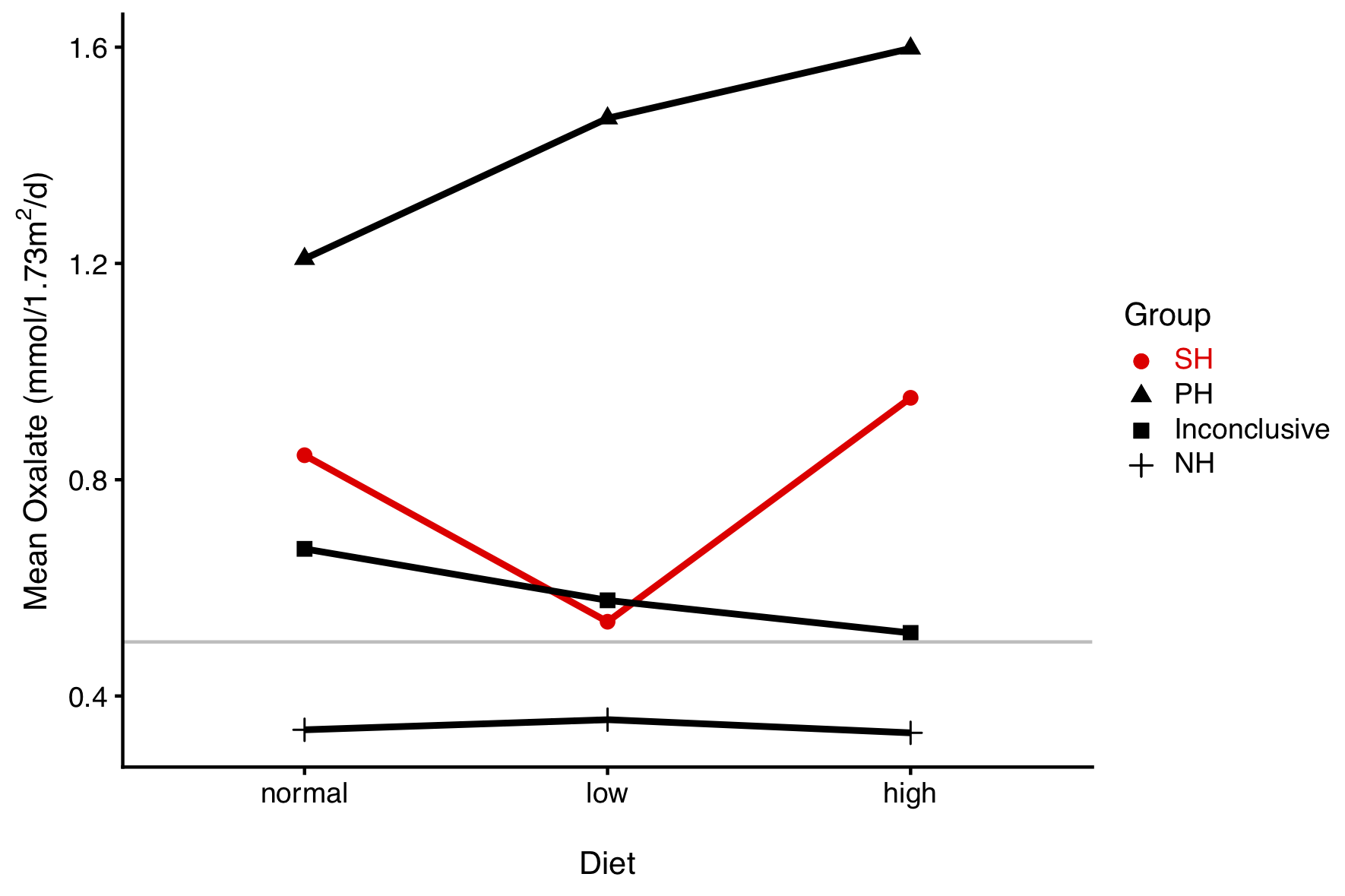

Supplement: Supplementary file 5 — Supplementary S-Fig. 1 Descriptive Plot showing the mean oxalate values of all groups under usual/normal, low and high oxalate diet. The horizontal line marks the hyperoxaluria threshold of 0.5 mmol/1.73m2/d (group 1: secondary hyperoxaluria (SH), group 2: suspected primary hyperoxaluria (PH), group 3: inconclusive, group 4: no hyperoxaluria (NH)) (PNG 80 kb) [file 240_2021_1290_MOESM5_ESM.png]
